# Supplementary material for: Anomalous Salt Dependence Reveals an Interplay of Attractive and Repulsive Electrostatic Interactions in α-synuclein Fibril Formation
Source: QRB Discov. 2020 Aug 6;1:e2. doi: 10.1017/qrd.2020.7 (PMC10392692; doi:10.1017/qrd.2020.7)
Supplement: Supplementary file 1 [file qrdsup.zip › S2633289220000071sup001.pdf]

```
# Faunus example input file for v2.2, git revision fa15d50
# https://github.com/mlund/faunus
{% set pH = 5.5 %}     # solution pH
{% set Cs = 0.030 %}   # ionic strength [mol/l]
{% set debyelength = 3.04/Cs**0.5 %}
{% set Lx = 500 %}     # box/slit dimentions
{% set Lz = 500 %}
{% set eps = 0.05*2.5 %} # LJ interaction strength

temperature: 298
random: {seed: fixed}            # random number seed
geometry: {type: slit, length: [{{ Lx }},{{ Lx }},{{ Lz }}]}
mcloop: {macro: 10, micro: 1000} # number of MC steps - this is a short run

atomlist: # list of amino acid beads, protonated and deprotonated
    - ANK: { r: 2.0, eps: 0, dp: 2, mw: 1e9} # heavy anchoring atom
    - ASP  :  { q: -1, r: 3.6, mw: 110, dp: 2, eps: {{ eps }} }
    - HASP :  { q:  0, r: 3.6, mw: 110, dp: 2, eps: {{ eps }} }
    - CTR  :  { q: -1, r: 2.0, mw: 16,  dp: 2, eps: {{ eps }} }
    - HCTR :  { q:  0, r: 2.0, mw: 16,  dp: 2, eps: {{ eps }} }
    - GLU  :  { q: -1, r: 3.8, mw: 122, dp: 2, eps: {{ eps }} }
    - HGLU :  { q:  0, r: 3.8, mw: 122, dp: 2, eps: {{ eps }} }
    - TYR  :  { q: -1, r: 4.1, mw: 154, dp: 2, eps: {{ eps }} }
    - HTYR :  { q: 0,  r: 4.1, mw: 154, dp: 2, eps: {{ eps }} }
    - CYS  :  { q: -1, r: 3.6, mw: 103, dp: 2, eps: {{ eps }} }
    - HCYS :  { q: 0,  r: 3.6, mw: 103, dp: 2, eps: {{ eps }} }
    - HIS  :  { q: 0,  r: 3.9, mw: 130, dp: 2, eps: {{ eps }} }
    - HHIS :  { q: 1,  r: 3.9, mw: 130, dp: 2, eps: {{ eps }} }
    - NTR  :  { q: 0,  r: 2.0, mw: 14,  dp: 2, eps: {{ eps }} }
    - HNTR :  { q: 1,  r: 2.0, mw: 14,  dp: 2, eps: {{ eps }} }
    - LYS  :  { q: 0,  r: 3.7, mw: 116, dp: 2, eps: {{ eps }} }
    - HLYS :  { q: 1,  r: 3.7, mw: 116, dp: 2, eps: {{ eps }} }
    - ARG  :  { q: 0,  r: 4.0, mw: 144, dp: 2, eps: {{ eps }} }
    - HARG :  { q: 1,  r: 4.0, mw: 144, dp: 2, eps: {{ eps }} }
    - ALA  :  { q: 0,  r: 3.1, mw: 66,  dp: 2, eps: {{ eps }} }
    - ILE  :  { q: 0,  r: 3.6, mw: 102, dp: 2, eps: {{ eps }} }
    - LEU  :  { q: 0,  r: 3.6, mw: 102, dp: 2, eps: {{ eps }} }
    - MET  :  { q: 0,  r: 3.8, mw: 122, dp: 2, eps: {{ eps }} }
    - PHE  :  { q: 0,  r: 3.9, mw: 138, dp: 2, eps: {{ eps }} }
    - PRO  :  { q: 0,  r: 3.4, mw: 90,  dp: 2, eps: {{ eps }} }
    - TRP  :  { q: 0,  r: 4.3, mw: 176, dp: 2, eps: {{ eps }} }
    - VAL  :  { q: 0,  r: 3.4, mw: 90,  dp: 2, eps: {{ eps }} }
    - SER  :  { q: 0,  r: 3.3, mw: 82,  dp: 2, eps: {{ eps }} }
    - THR  :  { q: 0,  r: 3.5, mw: 94,  dp: 2, eps: {{ eps }} }
    - ASN  :  { q: 0,  r: 3.6, mw: 108, dp: 2, eps: {{ eps }} }
    - GLN  :  { q: 0,  r: 3.8, mw: 120, dp: 2, eps: {{ eps }} }
    - GLY  :  { q: 0,  r: 2.9, mw: 54,  dp: 2, eps: {{ eps }} }
    - H+   :  { implicit: True, activity: {{ 10**(-pH) }} } # protons are "implicit", i.e
. not present in the box

moleculelist:
    - peptide: # full asyn
        structure: {fasta: "nMDVFMKGLSKAKEGVVAAAEKTKQGVAEAAGKTKEGVLYVGSKTKEGVVHGVATVAEKTK
EQVTNVGGAVVTGVTAVAQKTVEGAGSIAAATGFVKKDQLGKNEEGAPQEGILEDMPVDPDNEAYEMPSEEGYQDYEPEAc", k: 3,
 req: 7}
    - hair:    # c-term
        structure: {fasta: "anGKNEEGAPQEGILEDMPVDPDNEAYEMPSEEGYQDYEPEAc", k: 3, req: 7}
insertmolecules:
    - peptide: {N: 1}
    - hair: {N: 208}

reactionlist:
    # implicit protons and salt particles
    - HCTR = CTR + H+: {pK: 3.6}
    - HASP = ASP + H+: {pK: 3.9}
    - HGLU = GLU + H+: {pK: 4.1}
    - HHIS = HIS + H+: {pK: 6.5}
    - HCYS = CYS + H+: {pK: 8.5}
    - HNTR = NTR + H+: {pK: 8.6}
    - HTYR = TYR + H+: {pK: 10.1}
```

```
    - HLYS = LYS + H+: {pK: 10.8}
    - HARG = ARG + H+: {pK: 12.5}

energy:
    - confine:               # attach hair to one wall by constraining the
        type: cuboid         # mass center to a virtual box.
        molecules: [hair]    # The VERY heavy ANK atom defines the mass center
        com: true
        low: [-1e6, -1e6, -1e6]
        high: [1e6,1e6,{{ -0.5*Lz }}]
        k: 3
    - constrain: {type: molecule, index: 0, property: end2end, range: [0, {{ 0.5*Lx }}]}
# do not sample very extended chains
    - bonded: {} # harmonic bond energy
    - nonbonded_coulomblj:
        openmp: [g2g, i2all]
        cutoff_g2g:
            default: 1e6
            hair hair: {{ 3*debyelength + 150 }}
        lennardjones: {mixing: LB}
        coulomb: {epsr: 80, type: yukawa, debyelength: {{ debyelength }}, cutoff: {{ 3*de
byelength }} }
moves:
    - rcmc: {repeat: 1}      # reactive ensemble for proton titration
    - moltransrot: {molecule: peptide, dp: 20.0, dprot: 1, repeat: 1} # translate/rotate
    - transrot:    {molecule: peptide, repeat: 1}                      # translate aa bead
s
    - pivot:       {molecule: peptide, dprot: 0.3, repeat: 1}          # pivot move
    - moltransrot: {molecule: hair, dp: 6, dprot: 0.2, repeat: 1, dir: [1,1,1]}
    - transrot:    {molecule: hair, repeat: 1}
    - pivot:       {molecule: hair, dprot: 0.5, repeat: 1}

analysis:
    # on-the-fly analysis
    - sanity: {nstep: 1}
    - chargefluctuations: {nstep: 100, molecule: hair, pqrfile: hair.pqr}
    - chargefluctuations: {nstep: 100, molecule: peptide, pqrfile: peptide.pqr}
    - reactioncoordinate: {nstep: 6, file: mu.dat, type: molecule, property: mu, index: 0
}
    - reactioncoordinate: {nstep: 6, file: muz.dat, type: molecule, property: mu_z, index
: 0}
    - reactioncoordinate: {nstep: 6, file: zdist.dat, type: molecule, property: com_z, in
dex: 0}
    - reactioncoordinate: {nstep: 6, file: angle.dat, type: molecule, property: angle, in
dex: 0, dir: [0,0,1]}
    - reactioncoordinate: {nstep: 6, file: e2e.dat, type: molecule, property: end2end, in
dex: 0}
    - multipole: {nstep: 20}
    - systemenergy: {file: energy.dat, nstep: 500}
    - xtcfile: {file: traj.xtc, nstep: 5000}
    - qrfile: {nstep: 5000}
    - savestate: {file: confout.pqr}
    - savestate: {file: state.json}
```
