# Supplementary material for: Anomalous Salt Dependence Reveals an Interplay of Attractive and Repulsive Electrostatic Interactions in α-synuclein Fibril Formation
Source: QRB Discov. 2020 Aug 6;1:e2. doi: 10.1017/qrd.2020.7 (PMC10392692; doi:10.1017/qrd.2020.7)
Supplement: Supplementary file 1 [file qrdsup.zip › S2633289220000071sup002.pdf]

Anomalous salt dependence reveals an interplay of attractive and repulsive electrostatic interactions in α-Synuclein fibril formation

Ricardo Gaspar[1,2], Mikael Lund[3], Emma Sparr[1] and Sara Linse[2]

Departments of Physical-Chemistry[1], Biochemistry and Structural Biology[2] and Theoretical-Chemistry[3], Lund University, Lund, Sweden

Supporting Information

The aggregation of α-syn is pH dependent

Increasing pH leads to a change in slope with overall slower aggregation rates ultimately leading to undetectable aggregation within the time frame of the experiment. This pH dependence observed can be related to the deprotonation of both aspartic acid and glutamic acid residues highly concentrated in the C-terminal tail region. Their side chains have carboxylic acidic groups with low pK_a values. Therefore, increasing the pH would lead to an increase of the overall net negative charge of α-syn resulting in repulsion between monomer and existing seeds inhibiting the aggregation kinetics. For all cases studied the pH at which secondary nucleation is no longer effective, is in the range of 5.6 to 5.9. The same pH dependency previously reported using phosphate buffer was obtained for MES buffer, implying that it is independent to salt nature (Buell et al. 2014).

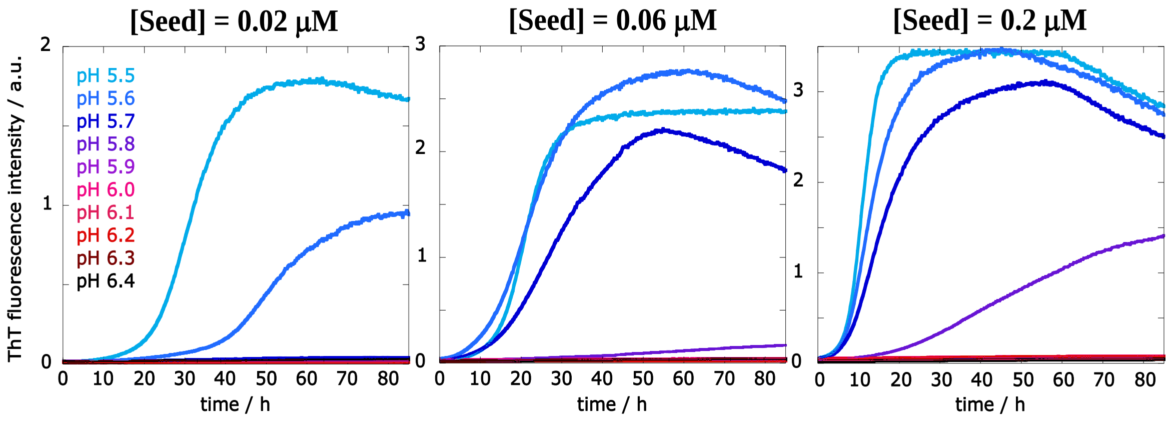

Figure S1. pH dependence on the aggregation kinetics of α-syn at low ionic strength. Aggregation kinetics starting from 20 µM α-syn monomer was monitored varying the pH from 5.5 to 6.4 in 10 mM MES buffer for three different seed concentrations at 37ºC and under quiescent conditions. The figure shows average traces of at least three experimental repeats.

The aggregation of α-syn is dependent on ionic strength

Aggregation kinetics were performed under quiescent conditions using non-binding PEGylated plates in 10 mM MES buffer pH 5.5 with added 140 mM NaCl for a range of monomer concentrations, 0.5-30 µM in the presence of four different seed concentrations varying from 0.003 to 3 µM. Aggregation was undetected in all conditions during the time frame of the experiment. Important to highlight, the slight ThT fluorescence increase seen in the panel correspondent to 3 µM seed is in fact only due to the contribution of the high amount of added seeds.

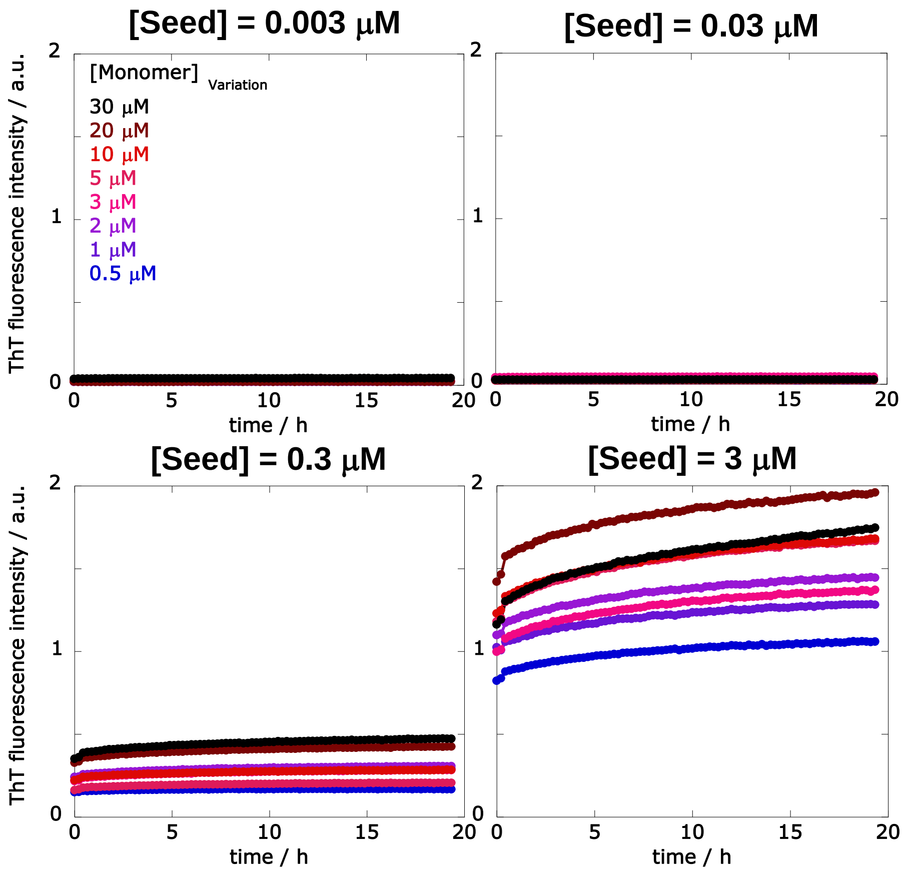

Figure S2. Seeded thioflavin-T aggregation kinetics of α-syn. Aggregation kinetics of α-syn monomer concentration variation ranging from 0.5–30 μM was monitored in the presence of different seed concentrations. Experiments were performed in 10 mM MES buffer pH 5.5 + 140 mM NaCl at 37ºC under quiescent conditions. The figures show averages of at least three traces as solid lines.

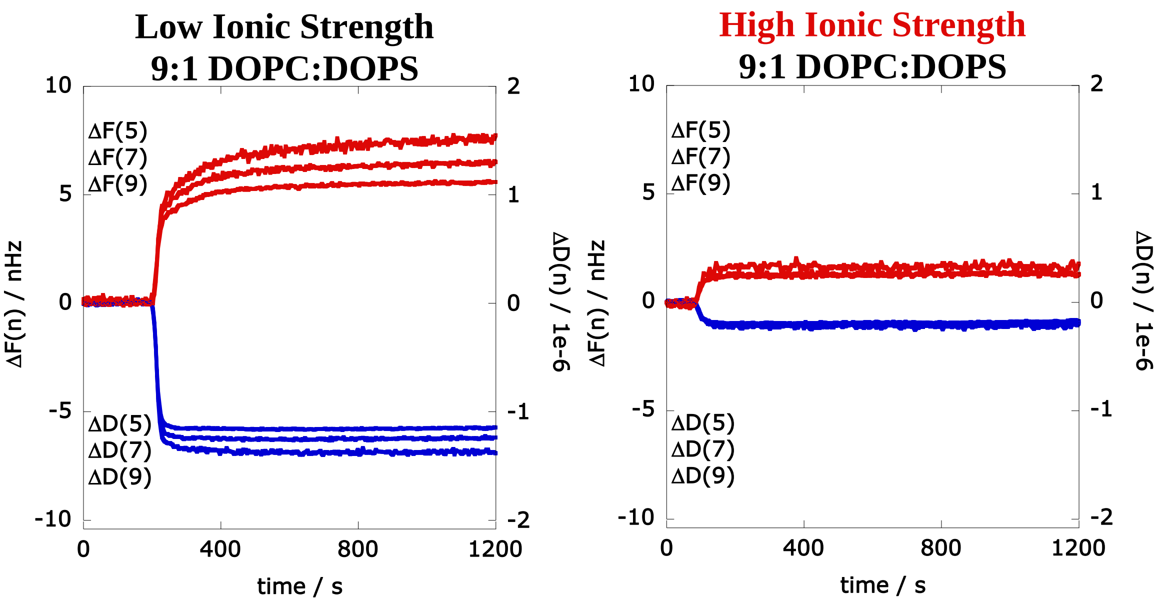

Figure S3. Adsorption of α-syn to negatively charged DOPC:DOPS (molar ratio 9:1) supported lipid bilayers at SiO_2 coated quartz crystals. Experiments were performed at mildly acidic pH (10 mM MES buffer, pH 5.5) without and with 150 mM NaCl. Shifts in frequency (blue) and dissipation (red) were monitored with QCM-D after injecting 4 μM α-syn to the solution that is in contact with the supported lipid bilayers. Each experimental condition was repeated at least three times.

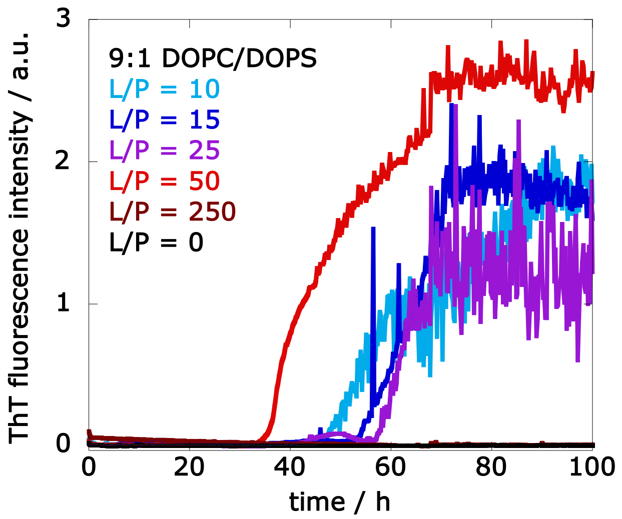

S4. Aggregation kinetics of α-syn in the presence of anionic lipid vesicles. Aggregation kinetics starting from 20 μM α-syn monomer was monitored by ThT in the presence of 9:1 DOPC/DOPS anionic lipid vesicles, in 10 mM MES buffer pH 5.5 at 37ºC under quiescent conditions. Thick lines represent the average traces of at least two experimental repeats.

Supplementary Method

Quartz Crystal Microbalance – Dissipation Measurements

A Q sense E4 instrument (Gothenburg, Sweden) and quartz crystals covered by a thin gold film coated with 50 nm SiO_2 (QSX 303, Q-sense) were used to perform the QCM-D measurements. The crystals had a fundamental frequency of 4.95 MHz. The experimental temperature was 25°C. The quartz crystals were stored in 2% SDS solution. The crystals before use were rinsed with deionized water and ethanol, dried with nitrogen and finally treated in a plasma cleaner (model PDC-3XG, Harrick Scientific Corp., Pleasantville, NY) under reduced air pressure for 5 minutes. A peristaltic pump (Ismatec IPC-N4) controlled the flow through in the four measuring cells. After placing the crystals in the instrument, stable baselines for the frequency and dissipation were achieved flowing water through the cells. The vesicle dispersions in aqueous solution with 100 mM NaCl were passed through the measuring cells at a flow rate of 100 µl/min for 10 minutes. After this period, the NaCl aqueous solution was replaced with water and then equilibrated with the desired experimental buffer (10 mM MES pH 5.5 or 10 mM MES pH 5.5 and 150 mM NaCl) prior to protein injection. The protein was diluted to a concentration of 4 µM, injected at a flow rate of 50 µL/min. For most of the experiments the total time of protein injection was approximately 20 minutes.

References

Buell AK, Galvagnion C, Gaspar R, Sparr E, Vendruscolo M, Knowles TPJ, Linse S and Dobson CM (2014). Solution conditions determine the relative importance of nucleation and growth processes in α-synuclein aggregation. *Proc. Natl. Acad. Sci. U.S.A.* **111**, 7671-7676.
